# Meat Eating

**Published:** 1898-02

**Authors:** 


### Meat-Eating.

Three popular errors in living, says Dr. Charles W. Purdy in the *North American Review*, consist in too much meat eating, an excessive consumption of sweet and starchy foods and over-eating. Of the latter he says: To the lay mind nothing seems to augur so strongly in favor of robust health as a hearty appetite. Furthermore, there would seem to be a strong conviction in the public mind, sanctified by tradition from time almost immemorial, that the more a man eats the better he is. The quantity of food that many people naturally eat is very large as compared with their actual physiological requirements; add to this the many tempting forms in which food is presented to the palate by our modern culinary arts, the sharpening of the appetite by the ante-prandial cocktail, the stimulus afforded the appetite by a bottle of good wine, and the result is often the consumption of an amount of food that simply overwhelms the assimilative organs. Such indulgence, if unrestricted and habitual, taxes both the assimilative and the excretory organs to their highest capacity, especially when coupled with sedentary life, and moreover it lends an additional impetus to the evils springing from the use of improper quality of food. The human elaborating and excretory mechanism was evidently adjusted for ordinary wear and tear to an average limited period of about seventy years. Under forty percent of extra work we must naturally expect impairment or breakdown of the mechanism much earlier. It should, therefore, excite no special surprise that so large a proportion of our well-to-do people die from Bright's disease, heart failure and allied diseases at fifty or fifty-five, who should, and under properly regulated lives and habits would, have attained the natural ages of seventy or over.

